# Mecânica Ventricular Esquerda: Desvendando as Vias da Resposta Cardiovascular ao Exercício

**DOI:** 10.36660/abc.20230181

**Published:** 2023-04-12

**Authors:** Eduardo M. Vilela, Ricardo Fontes-Carvalho

**Affiliations:** 1 Serviço de Cardiologia Centro Hospitalar de Vila Nova de Gaia/Espinho Gaia Portugal Serviço de Cardiologia, Centro Hospitalar de Vila Nova de Gaia/Espinho, Gaia – Portugal; 2 Centro de Investigação Cardiovascular Faculdade de Medicina Universidade do Porto Porto Portugal (UniC@RISE) Centro de Investigação Cardiovascular (UniC@RISE), Faculdade de Medicina, Universidade do Porto, Porto – Portugal

**Keywords:** Doenças Cardiovasculares/prevenção e controle, Doença Arterial Coronariana, Diagnóstico por Imagem/métodos, Ecocardiografia/métodos

A doença cardiovascular (DCV) é uma das principais causas de morbimortalidade, sendo a doença arterial coronariana (DAC) uma de suas apresentações mais desafiadoras.^1^ Ao longo dos anos, diversas estratégias permitiram melhorias marcantes em sua gestão. O exercício físico (um componente central dos programas de reabilitação cardíaca) é atualmente uma pedra angular das estratégias contemporâneas abrangentes de prevenção secundária.^2^ Embora os benefícios do exercício físico tenham sido extensivamente descritos na presença de DAC, as inúmeras vias mecanísticas pelas quais essa intervenção exerce seus diversos efeitos não foram totalmente elucidadas.^3 , 4^ De fato, embora seu impacto positivo na capacidade funcional tenha sido destacado, o papel relativo do treinamento físico na função sistólica mostrou alguma variabilidade em diferentes estudos.^4 , 5^Sem deixar de ter em consideração o fato de as diferenças nos desenhos dos programas e das populações em estudo poderem, pelo menos em parte, ajudar a explicar algumas dessas divergências, o estudo dos mecanismos pelos quais o exercício pode ter impacto na função cardíaca (nomeadamente no contexto da DAC) continua a ser um tema de grande interesse.

Avanços nas modalidades de imagem têm permitido uma compreensão cada vez mais precisa da fisiologia da DCV e do impacto de diferentes processos patológicos.^6 , 7^ Embora a fração de ejeção (FE) continue sendo o parâmetro mais onipresente para avaliar a função sistólica do ventrículo esquerdo (VE), outros parâmetros, como os derivados da imagem de deformação, têm sido cada vez mais destacados para tentar resolver algumas das armadilhas relacionadas à FE.^6 , 8 , 9^ A esse respeito, a noção de que diferentes orientações das fibras e sua interação complexa e dinâmica podem ser a chave para a função do VE levou progressivamente ao conceito de que o desenho estrutural do coração tem um papel particularmente central em sua biomecânica e, portanto, em sua função geral.^9 , 10^ Isto também enfatizou a possível relevância de parâmetros como o strain longitudinal global (GLS) e medidas auxiliares, como a torção e o twist do VE.^6 , 10^ Conforme detalhado anteriormente, eles podem permitir insights sobre os mecanismos de acoplamento entre sístole e diástole e função cardíaca global.^6 , 9 , 10^

Nesse contexto, Lima et al.,^11^ fornecem dados interessantes sobre a resposta da DCV ao treinamento físico em um grupo de pacientes após um infarto agudo do miocárdio (IM).^11^ Neste elegante estudo, sobreviventes de IM agudo (Killip classes I ou II) que tinham FEVE > 40% e que não relataram praticar atividade física regular antes do evento DCV foram randomizados para um programa de treinamento físico supervisionado (duas vezes por semana durante quatro meses) ou um grupo controle. Posteriormente, os pacientes foram submetidos a uma avaliação DCV detalhada, incluindo teste de esforço cardiopulmonar e ecocardiografia, incorporando speckle tracking.^11^ Nesta análise, abrangendo 53 pacientes (principalmente homens, 57% com hipertensão prévia, 30% diabéticos), aqueles randomizados para treinamento físico tiveram melhorias significativas na capacidade funcional durante o período de estudo, como atestado por aumentos no pico de consumo de oxigênio.^11^ Em termos de parâmetros ecocardiográficos, não houve diferenças entre o início e o final do estudo na FEVE em nenhum dos grupos. Notavelmente, o mesmo foi observado em termos de GLS.^11^ Conforme habilmente aludido pelos autores, ainda existem alguns obstáculos sobre os efeitos do treinamento físico no GLS, pois, embora os dados concordem com os achados atuais, também há relatos de um potencial benefício do exercício em termos de GLS.^11 - 13^ Quanto à FE, também neste contexto, as diferenças entre os desenhos (nomeadamente ao nível dos protocolos de treino e das características basais dos doentes) devem ser ponderadas.^12 , 13^ Curiosamente, no presente estudo, uma diferença significativa entre os grupos foi relatada na rotação basal, velocidade de twist e torção.^11^ Conforme reconhecido, essa redução na velocidade de twist também foi relatada em um estudo não randomizado em pacientes do sexo masculino após IAM submetidos a intervenção coronária percutânea.^14^ Embora, conforme habilmente discutido pelos autores, várias limitações potenciais, como o número de indivíduos em estudo, bem como questões técnicas relacionadas à avaliação de strain, devem ser ponderados, mas esses achados fornecem um importante incremento à literatura atual no campo, expandindo ainda mais a base de evidências para estudos futuros sobre esse tópico.^11 , 14 , 15^

O treinamento físico tem um papel primordial no continuum da DAC.^2 , 4 , 5^ À medida que a medicina cardiovascular continua a evoluir para fornecer uma abordagem cada vez mais personalizada para o paciente individual, em uma era que reflete a crescente complexidade e heterogeneidade da DAC, os dados derivados de diferentes modalidades sobre os efeitos do treinamento físico no coração podem fornecer informações importantes na busca interminável para uma maior personalização desta intervenção fundamental.
