# Supplementary material for: Mecânica Ventricular Esquerda: Desvendando as Vias da Resposta Cardiovascular ao Exercício
Source: Arq Bras Cardiol. 2023 Apr 12;120(4):e20230181. [Article in Portuguese] doi: 10.36660/abc.20230181 (PMC10263430; doi:10.36660/abc.20230181)
Supplement: Translation

# Translation: Left Ventricular Mechanics: Untwisting the Pathways of the Cardiovascular Response to Exercise

**Authors:** Eduardo M. Vilela, Ricardo Fontes-Carvalho

**Affiliations:** 1 Portugal Serviço de Cardiologia, Centro Hospitalar de Vila Nova de Gaia/Espinho, Gaia – Portugal; 2 Portugal Centro de Investigação Cardiovascular (UniC@RISE), Faculdade de Medicina, Universidade do Porto, Porto – Portugal

**Keywords:** Cardiovascuar Diseases/prevention and control, Coronary Artery Disease, Diagnostic Imaging/methods, Echocardiography/methods

Cardiovascular (CV) disease is a major cause of morbidity and mortality, with coronary artery disease (CAD) being one of its most challenging presentations.^1^O *v* er the years, several strategies have allowed marked improvements in its management. Physical exercise (a core component of cardiac rehabilitation programs) is currently a cornerstone of comprehensive contemporary secondary prevention strategies.^2^While the benefits of physical exercise have been extensively described in the presence of CAD, the myriad mechanistic pathways by which this intervention exerts its various effects have yet to be fully ascertained.^3 , 4^Indeed, while its positive impact on functional capacity has been highlighted, the relative role of exercise training in systolic function has shown some variability across different studies.^4 , 5^While considering that differences in program designs and populations under study could, at least partially, help explain some of these divergences, the study of the mechanisms by which exercise could impact cardiac function (namely in the setting of CAD) remains a topic of considerable interest.

Advances in imaging modalities have allowed for an ever more precise understanding of CV physiology and the impact of different pathological processes.^6 , 7^While ejection fraction (EF) remains the most ubiquitous parameter to assess left ventricular (LV) systolic function, other parameters, such as those derived from deformation imaging, have increasingly come under the spotlight to try to address some of the pitfalls related to EF.^6 , 8 , 9^In this regard, the notion that different fiber orientations and their complex and dynamic interplay could be key to LV function has progressively led to the concept that the heart’s structural design has a particularly central role in its biomechanics and, thus, its overall function.^9 , 10^These have also underscored the possible relevance of parameters such as global longitudinal strain (GLS) and ancillary measures such as LV twist and torsion.^6 , 10^As previously detailed, these could allow insights into the coupling mechanisms between systole and diastole and global cardiac function.^6 , 9 , 10^

In this background, Lima et al. provide interesting data on the CV response to exercise training in a group of patients after an acute myocardial infarction (MI).^11^In this elegant study, acute MI survivors (Killip classes I or II) who had an LVEF >40% and who did not report performing regular physical activity prior to the CV event were randomized to a supervised exercise training program (twice a week for four months) or a control group. Subsequently, patients underwent a detailed CV assessment, including cardiopulmonary exercise stress testing and echocardiography, incorporating speckle tracking.^11^In this analysis encompassing 53 patients (mostly male subjects, 57% with prior hypertension, 30% diabetics), those randomized to exercise training had significant improvements in functional capacity over the study period, as attested to by increases in peak oxygen consumption.^11^Regarding echocardiographic parameters, no differences between the study’s beginning and end were present on LVEF in either group. Notably, the same was observed in terms of GLS.^11^As expertly alluded to by the authors, there are still some hindrances concerning the effects of exercise training in GLS, as while data concurs with the current findings, there are also reports of a potential benefit of exercise in terms of GLS.^11 - 13^As for EF, also in this setting, differences between designs (namely in terms of training protocols and baseline patient characteristics) should be pondered.^12 , 13^Interestingly, in the present study, a significant difference between groups was reported in basal rotation, twist velocity, and torsion.^11^As acknowledged, this reduction in twist velocity has also been reported in a non-randomized study on male patients after a MI who had undergone percutaneous coronary intervention.^14^While, as expertly discussed by the authors, several potential limitations, such as the number of individuals under study as well as technical issues related to strain assessment, should be pondered, these findings nonetheless provide an important increment to the current literature on the field, further expanding the evidence base for future studies on this topic.^11 , 14 , 15^

Exercise training has a paramount role across the continuum of CAD.^2 , 4 , 5^As CV medicine continues to evolve to provide an increasingly tailored approach to the individual patient, in an era reflective of the growing complexity and heterogeneity of CAD, data derived from different modalities into the effects of exercise training on the heart could provide important insights on the unending quest for further personalization of this pivotal intervention.
